# A mini-whole lung lavage to treat autoimmune pulmonary alveolar proteinosis (PAP)

**DOI:** 10.1186/s12931-022-01982-2

**Published:** 2022-03-17

**Authors:** Francesca Mariani, Elena Salvaterra, Sara Lettieri, Annalisa De Silvestri, Alessandra Corino, Matteo Bosio, Elia Fraolini, Davide Piloni, Giuseppe Rodi, Angelo Guido Corsico, Ilaria Campo

**Affiliations:** 1 Pneumology Unit, IRCCS San Matteo Hospital Foundation, Pavia, Italy; 2 Department of Internal Medicine and Therapeutics, University of Pavia, Pavia, Italy; 3 Interventional Pneumology Unit, IRCCS University Hospital Sant’Orsola Malpighi, Bologna, Italy; 4 Clinical Epidemiology and Biometry Service, IRCCS San Matteo Hospital Foundation, Pavia, Italy; 5 Intensive Care Unit- IRCCS San Matteo Hospital Foundation, Pavia, Italy; 6 Pneumology Unit, Internal Medicine and Infectious Diseases Department, IRCCS Policlinico San Matteo Foundation, Pavia, Italy

**Keywords:** Pulmonary alveolar proteinosis, Whole lung lavage, ICU stay

## Abstract

**Background:**

PAP is an ultra-rare respiratory syndrome characterized by the accumulation of surfactant within the alveoli. Whole lung lavage (WLL) is the current standard of care of PAP, however it is not a standardized procedure and the total amount of fluid used to wash each lung is still debated. Considering ICU hospitalization associated risks, a “mini-WLL” with anticipated manual clapping and reduced total infusion volume and has been proposed in our center.

The aim of the study is to retrospectively analyze the efficacy of mini-WLL compared to standard WLL at the Pavia center.

**Methods:**

13 autoimmune PAP patients eligible for WLL were included: 7 patients were admitted to mini-WLL (9 L total infusion volume for each lung) and 6 patients underwent standard WLL (14 L of infusion volume). Functional data (VC%, FVC%, TLC%, DLCO%) and alveolar-arterial gradient values (A-aO2) were collected at the baseline and 1, 3, 6, 12, 18 months after the procedure.

**Results:**

A statistically significant improvement of VC% (p = 0.013, 95%CI 3.49–30.19), FVC% (p = 0.016, 95%CI 3.37–32.09), TLC% (p = 0.001, 95%CI 7.38–30.34) was observed in the mini-WLL group in comparison with the standard WLL group, while no significant difference in DLCO% and A-aO2 mean values were reported.

**Conclusion:**

Mini-WLL has demonstrated higher efficacy in ameliorating lung volumes, suggesting that a lower infusion volume is sufficient to remove the surfactant accumulation and possibly allows a reduced mechanical insult of the bronchi walls and the alveoli. However, no statistically significant differences were found in terms of DLCO% and Aa-O2.

## Introduction

Pulmonary alveolar proteinosis (PAP) is an ultra-rare respiratory syndrome characterized by accumulation of surfactant within pulmonary alveoli resulting in a variable impairment of pulmonary gas transfer, until hypoxemic respiratory failure and death [1–3]. Recent achievements in the knowledge of PAP pathogenesis have opened to novel pathogenesis-based therapeutic approaches, the most promising ones are therapies targeting GM-CSF pathway [4, 5]. The results of the largest study to date, the phase II/III, randomized, double-blind, placebo-controlled multicenter clinical trial (IMPALA study), have been lately released [9]. IMPALA study has investigated the efficacy and safety of inhaled recombinant GM-CSF (Molgramostim, Savara Aps) in 138 patients reporting an improvement in the alveolar-arterial difference in oxygen concentration (A-aDo_2_) in the treated group compared to the placebo group, with similar rates of adverse events. Despite these promising results, whole lung lavage (WLL) remains the gold standard treatment [6]. WLL improves symptoms, functional and radiological abnormalities and oxygenation with an immediate positive outcome in > 90% of patients, even if a recurrence rate ranging from 30 to 70% has been reported, with the need for repeated lavages [2, 7–9]. WLL is usually performed under general anaesthesia with a double-lumen tracheal tube. While mechanical ventilation is maintained in one lung, the contralateral one undergoes repeated cycles of instillation of saline warmed at 37 °C (up to 50 L per lung) and drainage by gravity, associated with chest percussion to emulsify the surfactant sediment. The manual clapping generally starts after the first 4–6 L of instilled saline and continues until the lavage fluid becomes clear [7, 10, 11].

WLL procedure, indications for its execution, contraindications and criteria to measure outcomes have not been standardized and international consensus documents are lacking. WLL is performed in a limited number of highly specialized centres whose expertise is the result of a continuous process of self-apprenticeship and strong cooperation of skilled pulmonologists and anaesthetists. To reduce complications of the procedure and risks related to the prolonged alveolar flooding, in our center we proposed a “mini-WLL” procedure with anticipated manual clapping of the chest and, consequently, a reduced infusion volume for each lung, from 15–20 to 9 L. The present study aims to retrospectively analyze the efficacy of the mini-WLL compared to the standard procedure.

## Methods

### Study design

We retrospectively analyzed 13 autoimmune PAP adult patients referred to our center and admitted to WLL treatment. PAP syndrome diagnosis was made according to the typical chest high-resolution CT findings (HRCT), “crazy paving pattern”, and the presence of opaque, milky bronchoalveolar lavage fluid (BALF) and/or the presence of amorphous, eosinophilic, PAS-positive material, as well as lipid-laden macrophages on bronchoalveolar lavage analysis [12]. All the patients underwent medical history collection, physical examination, laboratory tests, anti-GM-CSF autoantibodies (GMAbs) dosage, radiological assessment with double projection chest X-Ray and HRCT, arterial blood gas analysis, global spirometry, CO diffusion capacity test, exercise tolerance testing (modified Bruce protocol or 6mWT) and broncho-alveolar lavage (BAL) cytology. Determination of serum level of GMAbs was performed in the Laboratory of the Rare Lung Disease Consortium at the Cincinnati Children’s Hospital Medical Center. Serum biomarkers measurements (LDH, Cyfra 21-1, CEA, NSE) were performed in the clinical chemistry facilities according to internal standard operative procedures.

From 2009 to 2011, six autoimmune PAP patients (control group) underwent standard WLL, seven patients (study group) were admitted to “mini-WLL” from 2014 to 2016.

The “mini-WLL” procedure is characterized by the starting of chest percussion in concomitance with the first aliquot infusion and, consequently, consists in a reduction of the infusion volume for each lung from 15–20 to 9L.

Although there are no clearly established criteria for performing WLL, the recommendations followed by the Pavia Center include: (i) presence of persistent or progressive respiratory failure; (ii) absence of respiratory difficulty at rest, but drop by 5 or more percentage points of O_2_ saturation on exercise tolerance test (modified Bruce protocol) determined by pulse oximetry; (iii) in selected cases, WLL may be discussed if a PAP patient, in particular a young adult, reports a significant limitation in daily or sports activities.

One month before the WLL and 1, 3, 6, 12, 18 months after the treatment pulmonary function tests were performed. The study also considered, in the period of observation (18 months), if there was a relapsing disease in need for a new WLL procedure.

The investigation was conducted in compliance with the Helsinki declaration.

### Statistical analysis

Qualitative variables were summarized as counts and percentages; quantitative variables were described as mean values and standard deviations. Respiratory parameters during the observation period were analyzed by fitting multilevel (time and patients) mixed-effect regression models in which the random portion consisted in patients (thus correcting for individual variation) and the fixed portion were represented by time and groups while age, sex, and smoking history were the confounding factors. The model is a random intercept allowing each subject to have a separate intercept; thus it is possible to take into account the dependency of the data (obtaining the correct standard errors) and to adjust for individual factors (measured and unmeasured confounders)The model assumes a constant correlation between all observations on the same subject. The analysis objectives was to measure the average treatment effect over time through coefficient beta and 95% confidence interval (95%CI). P < 0.05 was considered statistically significant. Data analysis was performed with STATA v16.1 (Stata Corporation, College Station, Texas, USA).

## Results

From 2009 to 2016, 13 autoimmune PAP patients underwent WLL. Demographic, clinical, and assessment features of the patients included in the study are reported in Table 1.

**Table 1:** Characteristics and assessment of the PAP cohort

	Study group [n = 7]	Control group [n = 6]	
Age, years [mean ± SD]	42.7 ± 16	53.3 ± 9.4	
Ratio male/female	4/3	3/3	
Smoking status [n (%)]			
Current	1 (14.28%)	1 (16.66%)	
Previous	2 (28.57%)	3 (50.00%)	
Never	4 (57.14%)	2 (33.33%)	
Symptoms at diagnosis [n (%)]			
Dyspnoea	5 (71.42%)	5 (83.33%)	
Cough	5 (71.42%)	4 (66.66%)	
Fever	2 (28.57%)	–	
Fatigue	2 (28.57%)	–	
Biomarkers [mean ± SD]			
Cyfra 21.1 (ng/mL)	11.85 ± 8.7	16 ± 11.26	(nv: 0.0–3.3)
CEA (ng/mL)	13.71 ± 13.46	18 ± 20.07	(nv: 0.0–5.0)
NSE (ng/mL)	24 ± 11.60	21.33 ± 14.19	(nv: 0.0–15.0)
GMAbs (μg/mL)	108.44 ± 80.33	216.20 ± 263.63	(nv < 3)
Infection at diagnosis [n (%)]	4 (57.14%)	3 (50.00%)	
Time to diagnosis, months [mean (range)]	7 (1–23)	4 (0–16)	
1° lung flooding time, minutes [mean ± SD]	34 ± 8	50 ± 8	
2° lung flooding time, minutes [mean ± SD]	29 ± 2	48 ± 9	

*Cyfra 21.1* cytokeratin fragment 21.1; *CEA* carcinoembryonic antigen; *NSE* neuron-specific enolase; *GMAbs* autoantibodies anti GM-CSF, *SD* standard deviation; *nv* normal value

The study group was composed of 7 patients (mean ± SD GMAbs = 108.44 ± 80.33), 4 males and 3 females. At the time of WLL, the mean ± SD age was 42.7 ± 16 years. Smoking history was reported in three patients (one current and two former) while four patients were never smokers.

Among symptoms reported at the time of diagnosis, the majority of PAP patients complained about dyspnoea (71.4%) and cough (71.4%), only one patient reported fever together with dyspnoea and fatigue. The mean interval between symptoms onset and diagnosis was 7 months. Serum biomarkers measured at the time of WLL showed a mean value above the reference upper limit (mean ± SD Cyfra21.1 = 11.85 ± 8.7 ng/mL; CEA = 13.71 ± 13.46 ng/mL; NSE = 24 ± 11.60 ng/mL). Four patients were affected by a respiratory infection (*Pseudomonas aeruginosa*, *Mycoplasma pneumoniae*, *Staphylococcus aureus*, *Klebsiella pneumoniae*) at diagnosis.

In the study group, the first lung was flooded for 34 ± 8 min and the second lung for 29 ± 2 min (mean time).

The control group was composed of 6 patients (mean ± SD GMAbs = 216.20 ± 263.63), 3 males and 3 females. The mean ± SD age was 53.3 ± 9.4. At the time of WLL, one patient was current, three former and two never smokers. Among symptoms reported at the time of diagnosis, the majority of patients complained about dyspnoea (83.3%) and cough (66.7%). The mean interval between symptoms onset and diagnosis was 4 months. Also in the control group serum biomarkers measured at the time of WLL showed a mean value above the reference upper limit (mean ± SD Cyfra 21.1 = 16 ± 11.26 ng/mL; CEA = 18 ± 20.07 ng/mL; NSE = 21.33 ± 14.19 ng/mL). Three patients were affected by a respiratory infection (*Staphylococcus aureus*, *Pneumocystis jirovecii*, *Mycoplasma pneumoniae*) at the time of diagnosis.

In the control group, the first lung was flooded for 50 ± 8 min and the second lung for 48 ± 9 min (mean time).

As expected, at baseline, serum LDH levels were above the upper reference limit in all enrolled PAP patients (data not shown).

**Fig. 1:**
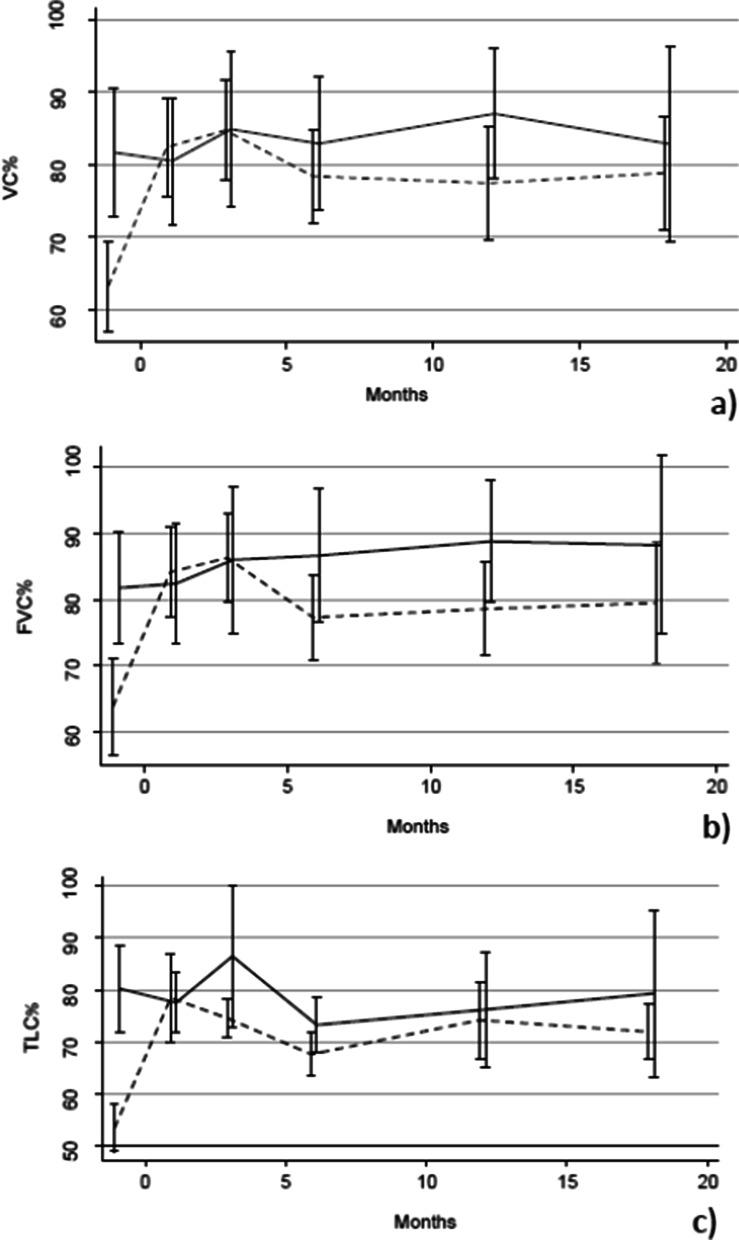
Pulmonary function test performed 1 month before the WLL and 3, 6, 12, 18 months after the treatment. **a** VC%, **b** FVC% and **c** TLC% of predicted volumes. Continuous line = mini-WLL; dotted line = standard WLL. Vertical bars represent confidence interval

For each patient admitted to WLL, lung function data were collected 1 month before the WLL and 1, 3, 6, 12, 18 months after the treatment. The mean values and standard deviations of alveolar-arterial gradient (A-aO2), vital capacity, forced vital capacity, total lung capacity, and carbon monoxide diffusion capacity measured as percentage of the predicted value (VC%, FVC%, TLC%, and DLCO%, respectively) were evaluated in the two groups. The multivariate regression analysis demonstrates a significant improvement during the whole follow up of VC% (beta = 16.84, p = 0.013, 95%CI 3.49–30.19) (Fig. 1a), FVC% (beta = 17.73, p = 0.016, 95%CI 3.37–32.09) (Fig. 1b), TLC% (beta = 18.86, p = 0.001, 95%CI 7.38–30.34) (Fig. 1c) in the study group compared to the control group.

Our data show a significant improvement in DLCO% (p < 0.001) and in A-aO2 (p = 0.005) mean value in all the treated patients during the period of observation, thus confirming the efficacy of mini-WLL. However, the multivariate regression analysis shows no statistically significant difference between the study and the control group regarding DLCO% (beta = 5.33, p = 0.413, 95%CI − 7.23 to 18.09) and A-aO2 (beta = − 2.97, p = 0.612, 95%CI − 14.46 to 8.51), respectively.

Progression of the disease requiring an additional WLL was reported in 4 patients (2 in the study group and 2 in the control group), 6 and 12 months after the treatment in both the two groups.

## Discussion

WLL is an invasive treatment, practiced under general anaesthesia and requiring prolonged hospitalization in intensive care unit. Although WLL is a safe procedure, complications are reported in about 18% of cases, including fever, hypoxemia, wheezing, pneumothorax, hydrothorax, minor bleeding from airway injury, balloon rupture, superimposed infection, and acute respiratory distress syndrome [13].

As a first step to standardizing WLL procedure, in 2016 a global survey, including 30 centres performing WLL in adults and paediatric patients, was conducted [13]. This survey highlighted a substantial heterogeneity among centres about indications and contraindications for the procedure and technical aspects, such as patient position, lung selection, total volume of lavage, chest percussion, thus providing an instrument for developing an international consensus document, in order to optimize safety and efficacy of the procedure.

From 2014 to 2016, a WLL procedure called “mini-WLL”, with anticipated manual clapping of the chest, reduced infusion volume, and lung flooding time, has been implemented in our center. In this retrospective study, we compared the mini-WLL (study group) with the standard procedure (control group), to clarify if the infusion volume could affect the efficacy of the lavage in terms of respiratory function. In particular, we analyzed the functional parameters most frequently impaired in PAP, such as VC%, FVC%, and TLC% of predicted volumes, which suggest the typical PAP restrictive ventilatory pattern. At the baseline, in the two groups of autoimmune PAP patients, we demonstrated as expected the reduction of lung volumes compared to predicted values, which indicates a respiratory restrictive disease.

The multivariate regression analysis shows a statistically significant improvement in term of mean values of VC%, FVC% and TLC% in the study group in comparison with the control group, thus suggesting that “mini-WLL” is more effective than the standard procedure on lung volumes recovery. Taking into account the reduced amount of saline infusion volumes for each lung used in the mini-WLL (9L vs 15-20L, in standard WLL), it is plausible to hypothesize that a lower infusion volume is sufficient to remove the surfactant accumulation within the alveoli. Furthermore, a shorter lung flooding time (about 1 h vs 1 h and a half, for both the lungs), could allow a reduced mechanical insult of the airway walls and the alveoli and a reduced local inflammatory response thus enabling a quicker recovery of the lung volumes. The procedure would also become safer, as potential complications such as spillover to the opposite lung, overdistension of the alveoli and systemic absorption of the saline solution would be reduced.

In our study population, DLCO% measured 1 month before the lavage is severely compromised and both the two WLL methods determine a significant improvement during the 18 months of observation. However, the comparison between the two groups shows no statistically significant difference. Accordingly, we can speculate that the mini-WLL is effective as the standard WLL in term of DLCO% amelioration, but it is not clear whether the mini-WLL enables a better gas exchange.

In both, the study and control groups, the alveolar-arterial gradient measured 1 month before the lavage indicates a ventilation-perfusion mismatch and an intra-pulmonary shunt, peculiarity frequently found in PAP patients eligible for WLL. During the observation period, the comparison of mean A-aO2 values between the two groups showed no statistically significant difference. Accordingly, we can theorize that the mini-WLL is effective as the standard WLL in term of A-aO2 amelioration, but if the mini WLL allows a better improvement in term of oxygen transfer from alveoli to blood remains an open question.

This study is limited by its retrospective nature that restricts the accessibility of complementary data, such as radiological findings. Enrolled participants cannot be made equal through random assignment, however the statistical analysis here performed adjusted for pre-existing differences in non-equivalent groups.

We cannot exclude that an increasing expertise in diagnostic reasoning process could have finally improved also the practice setting, even if no changes in supportive care was made between 2009 and 2016. This issue was also adjusted in the statistical analysis. Moreover, as LDH diagnostic assay changed in 2011 in our central laboratory, we cannot compare the serum levels measured before and after this date. The small sample size is another critical issue; however, the extreme rarity of PAP should be taken into consideration. Further analysis are thus necessary to confirm the results here presented.

## Conclusion

We have demonstrated the safety and efficacy of “mini-WLL”. Compared to standard lavage, the “mini-WLL” requires a shorter procedural time that means reduction in time of lateral decubitus position and general anesthesia. Therefore, the “mini-WLL” reduces the risk of complications and lowers hospitalization costs. In order to confirm these results, further evidences from randomized-controlled trials are required.

## Data Availability

The dataset used and analyzed during the current study available from the corresponding author on reasonable request.
